# A Dual-Band Conformal Antenna Based on Highly Conductive Graphene-Assembled Films for 5G WLAN Applications

**DOI:** 10.3390/ma14175087

**Published:** 2021-09-06

**Authors:** Zelong Hu, Zhuohua Xiao, Shaoqiu Jiang, Rongguo Song, Daping He

**Affiliations:** 1 Hubei Engineering Research Center of RF-Microwave Technology and Application, Wuhan University of Technology, Wuhan 430070, China; 54hushelong@whut.edu.cn (Z.H.); 17762546489@whut.edu.cn (S.J.); 2 School of Information Engineering, Wuhan University of Technology, Wuhan 430070, China; lorensteven@whut.edu.cn

**Keywords:** dual-band, graphene-assembled films, conformal antenna, Wi-Fi antenna, 5G

## Abstract

Flexible electronic devices are widely used in the Internet of Things, smart home and wearable devices, especially in carriers with irregular curved surfaces. Light weight, flexible and corrosion-resistant carbon-based materials have been extensively investigated in RF electronics. However, the insufficient electrical conductivity limits their further application. In this work, a flexible and low-profile dual-band Vivaldi antenna based on highly conductive graphene-assembled films (GAF) is proposed for 5G Wi-Fi applications. The proposed GAF antenna with the profile of 0.548 mm comprises a split ring resonator and open circuit half wavelength resonator to implement the dual band-notched characteristic. The operating frequency of the flexible GAF antenna covers the Wi-Fi 6e band, 2.4–2.45 GHz and 5.15–7.1 GHz. Different conformal applications are simulated by attaching the antenna to the surface of cylinders with different radii. The measured results show that the working frequency bands and the radiation patterns of the GAF antenna are relatively stable, with a bending angle of 180°. For demonstration of practical application, the GAF antennas are conformed to a commercial router. The spectral power of the GAF antenna router is greater than the copper antenna router, which means a higher signal-to-noise ratio and a longer transmission range can be achieved. All results indicate that the proposed GAF antenna has broad application prospects in next generation Wi-Fi.

## 1. Introduction

Flexible, low profile and conformal antennas have a wide application prospect in the fields of smart wearable devices, Internet of things (IoTs), implantable biological circuits, national defense industry and smart homes [1,2,3,4,5,6]. Furthermore, to adapt to the development trend of miniaturization, multi-function and integration of electronic devices, dual-band/multi-band antennas emerged at a historic moment [7,8,9,10] and can reduce the number of antennas, thus reducing the cost and size and ameliorate the electromagnetic compatibility problems. Romain Berges et al. proposed a printed wearable dual-band antenna for a wireless power transfer [11]. Using polyimide film as the dielectric substrate, the antenna has good flexibility, but bending has a great influence on its resonance characteristics. Mohammad Haerinia et al. proposed a printed conformal dual-band antenna for wireless energy harvester [12]. The designed antenna has a narrow bandwidth and low gain. These antennas have the advantages of convenient processing and easy to mass production, but the low conductivity of conductive ink limits the radiation performance. Niamat Hussain designed a multi-band flexible antenna for heterogeneous applications using the method of frequency reconfigurable [13], which has the advantages of wide impedance bandwidth and stable radiation pattern. However, using bias diodes increases the complexity of the circuit. Furthermore, the proposed copper-based dual-band antenna is easy to break after repeated bending, which is not suitable to apply in the scene of repeated bending such as smart wear. To solve these problems, there is an urgent need for the flexible dual-band antenna which can be bent repeatedly and has excellent radiation performance.

To overcome the drawbacks of metal materials of poor flexibility and easy to break, some new conductive materials have attracted increasing attention, such as metal mesh conductive sheet [14], conductive ink [15,16,17], and a carbon nanotube [18]. Nevertheless, the metal mesh conductive sheet is too expensive and prone to oxidation [14], the conductive ink normally has low conductivity which is not suitable for antenna design [15,16,17], and the carbon nanotube also has a relatively high sheet resistance, resulting from junction resistance and is not possible to produce large-area film [18]. Recently, graphene-assembled films with the advantages of high conductivity, good bending resistance and good chemical stability have been considered as a promising alternative conductive material for antenna [19,20,21], sensor [22,23,24] and other RF applications [25,26], which show great potential in overcoming the challenges of conformal antenna design [27,28,29]. It is worth noting that most of these flexible antennas are single-band and their bending angle is greatly limited. Moreover, the resonant characteristics of the antenna deteriorate and it cannot work properly after bending. Therefore, the research of flexible conformal antenna is still lacking, especially the multi-band antenna.

In this paper, a dual-band conformal antenna is proposed based on graphene assembled films (GAF) with a high conductivity of 1.13 × 10^6^ S/m. The operating frequency bands of flexible GAF antenna cover Wi-Fi 6e bands of 2.4–2.45 GHz and 5.15–7.1 GHz. The proposed GAF antenna has a low profile of 0.548 mm. Even the GAF antenna is bent with angle of 180°, the working frequencies are 2.33–2.45 GHz and 5.06–7.1 GHz, which can still meet the requirements. The measured maximum realized gain is 6.85 dBi at 6.12 GHz. The measured results show that the radiation patterns are relatively stable after bending. In addition, the GAF antennas are assembled in a commercial router for performance tests. The experimental results show that the GAF antenna router has a higher spectral power than the initial router. With the advantages of simple structure, low profile and good flexibility, the proposed conformal antenna has a broad application prospect in the fields of Internet of Things and intelligent wearables.

## 2. Materials and Methods

The graphene-assembled films are fabricated by the following four steps [29]: Firstly, graphene oxide (GO) slurry was diluted with ultrapure water to obtain the GO suspension with concentration of 15 mg/mL. Then, the GO suspension was scraped onto a polyethylene terephthalate (PET) substrate and then evaporated to obtain the GO assembly films. Thereafter, the GO assembly films were annealed at 1300 °C for 2 h and 3000 °C for 1 h in argon (Ar) gas flow. Finally, the GAF was achieved by a rolling compression process with the pressure of 150 MPa.

**Figure 1:**
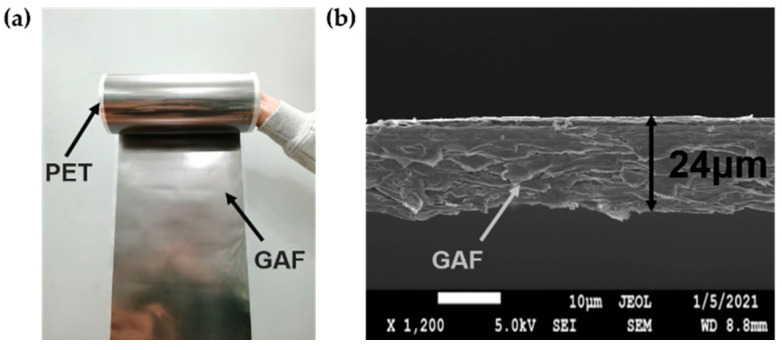
(**a**) Digital photo of the GAF. (**b**) Cross-sectional SEM image of the GAF.

The digital photo in Figure 1a shows that the GAF has good flexibility and can be arbitrarily bent to fit different conformal scenarios. The electrical conductivity of the graphene assembled films is 1.13 × 10^6^ S/m measured by using a four-point probe resistance measurement system. Moreover, the density of the GAF is 1.48 g/cm^3^, which is smaller than a quarter of the copper foil of 8.8 g/cm^3^. Figure 1b is the cross-sectional scanning electron microscopy (SEM) (JEOL JEM6700, Tokyo, Japan) image of the GAF showing the thickness of the GAF to be 0.024 mm.

## 3. Results and Discussion

### 3.1. Design and Simulation of GAF Dual-Band Antenna

**Figure 2:**
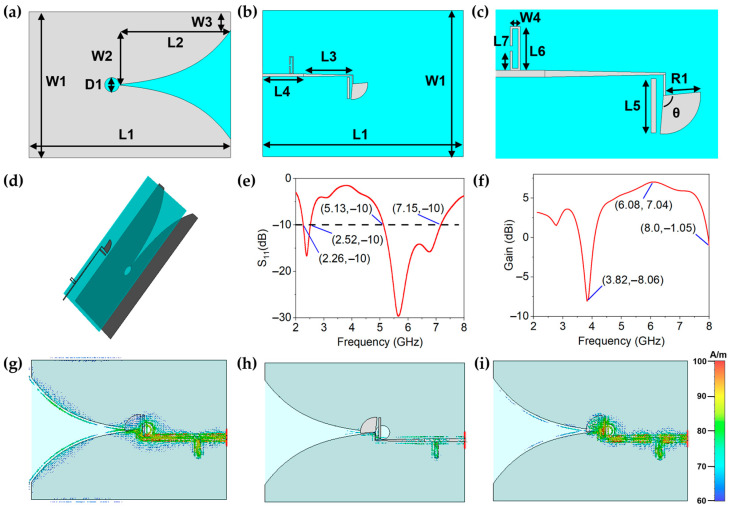
(**a**–**d**) Structure of the proposed flexible GAF dual-band antenna. (**a**) Top view. (**b**) Bottom view. (**c**) The model of feed structure. (**d**) Perspective view. (**e**) Simulated reflection coefficients of the GAF antenna under flat state. (**f**) Simulated gain of the GAF antenna under flat state. (**g**–**i**) Surface current distributions of the GAF antenna at 2.45 GHz, 3.8 GHz and 8 GHz.

To cover all frequency bands under the Wi-Fi 6e standard (2.4–2.45 GHz and 5.15–7.1 GHz), the dual-band antenna is designed in a sandwich structure, as shown in Figure 2a–d. The top and bottom layers are radiating conductors, which are made of graphene assembled films. As shown in Figure 2a–c, W1 is the width of the substrate, W2 is the width of the aperture, L1 is the length of the substrate, L2 is the length of the substrate, L3 is the length of the matching line, L4 is the length of the feed line, L5 is the length of the open circuit half wavelength resonator, D is the radii of the circular slot and R1 is the Radius of the fan coupling line. To achieve good conformal characteristics, flexible PDMS is chosen as the dielectric substrate (blue part) with thickness of 0.5 mm, dielectric constant of 2.7 and loss tangent angle of 0.02. Figure 2a,b depicts the structure of the proposed Vivaldi antenna, which is a broadband end-fire traveling wave type. It is usually implemented on a substrate with the Vivaldi design etched on the upper cladding of the substrate.

In order to realize dual-band radiation, band-notch structures are added to the ultra-wideband (UWB) antenna, which not only has the characteristic of low profile, but also can flexibly adjust the notch frequencies. Among the UWB antennas, Vivaldi antenna has the advantages of low profile, wide band, simple structure and stable radiation pattern. By adding notch structures beside the microstrip feeder, good stop-band suppression can be achieved, which has the advantages of simple structure and low profile. The basic structure of the Vivaldi antenna consists of a λ/4 uniform slot that is connected to an exponentially tapered slot. To reduce the return loss of antenna, the λ/4 uniform slot can be replaced by a circular slot with radius of λ/8. The slot is excited by a microstrip transmission line from the undersurface of the substrate. The Vivaldi design is usually of low cost, and it possesses excellent radiation characteristics, such as high gain, broadband performance, constant beam width, and low side lobes. Parametric studies show that the directivity of Vivaldi antennas increases as the length L2 of the antenna increases, and the bandwidth is influenced by the opening width W3 and the aperture width W2 of the antenna. It is also worthy to notice that as the length of the antenna increases, the beam width narrows.

The exponential taper curve is given by the following formula:

(1) $$y(x)=\pm Ae^{px}+B$$

where *y* is the half separation of the slot and *x* is the position across the length of the antenna, *A* is half of the opening width W3, *p* is the taper rate and *B* can be determined by W1, W2 and W3 (W1/2 = W2 + W3 + *B*). The initial value of parameter *B* is set as a quarter wavelength of the highest resonant frequency of the antenna, and then its optimal value is determined by means of parameter scanning. To improve the resistance at low frequency, the value of p can be increased. However, large values of *p* simultaneously create larger variations in the resistance and reactance over the entire band, which will worsen the resonant characteristics of the antenna. Therefore, a compromise is usually required between the taper rate *p* and the square resonant/cavity area to achieve wide bandwidth. In addition to the bandwidth, the taper rate also has a great impact on the antenna beam width. In general, the taper rate is positively correlated with the beam width in the E-plane, and inversely correlated with the beam width in the H-plane. Therefore, to obtain a wide beam width in the E-plane, the taper rate needs to be increased. Parametric studies have shown that the optimal performance is achieved when the length L2 is greater than one wavelength at the lowest frequency.

To obtain stable far-field radiation characteristics, the scheme of ultra-wideband and band-notch are adopted to achieve dual-frequency band. Firstly, a Vivaldi antenna with the advantages of frequency independence, simple structure and low profile is designed to achieve ultra-wide working band of 2.5–10 GHz. Then, two notch elements are added to trap the non-resonant frequencies to suppress its radiation. These two notch units are the split ring resonator (SRR) and the open circuit half wavelength resonator (OCHWR) where *f_n_*_1_ is the notch frequency of SRR and *f_n_*_2_ is the resonant frequency of OCHWR. SRR and OCHWR are widely used in band-notch antenna, which have the characteristics of simple structure, low profile and high flexibility [30,31]. In addition, double-capacitor load ring resonator (DCRR) [32], stub-loaded resonator (SLR) [33,34] and step impedance resonator (SIR) [35,36,37] can also be used as notch elements, although the design is more complex. The equivalent circuit of the SRR can be considered as a high-Q LC resonance tank, the splitting gap provides a capacitor (Cr) and the inductor (Lr) comes from the current loop [32]. By adjusting the value of Lr and Cr, the resonant frequency can be dynamically adjusted. In practical design, the desired resonant frequency is usually achieved by changing the total length of the resonator. The frequency of the resonator is negatively correlated with its total length, that is, when the total length increases, the resonant frequency decreases. In this paper, *f_n_*_1_ is 3.8 GHz and *f_n_*_2_ is 8 GHz. Then the length of SRR and OCHWR can be calculated by the following formulas:

(2) $$L_{SRR}=c/(2f_{n1}\sqrt{\varepsilon_{e}})$$

(3) $$L_{OCHWR}=c/(2f_{n2}\sqrt{\varepsilon_{e}})$$

where, c is the speed of light and *ε_e_* is the effective dielectric constant of the substrate.

The antenna is modeled and simulated in CST software based on the calculated parameters. As shown in Figure 2e, after optimization, the simulated resonant frequencies of the GAF dual-band antenna are 2.27–2.52 GHz and 5.13–7.17 GHz, and the reflection coefficients at 5.65 GHz is −30 dB, which indicates good impedance match of the antenna and the feeding system. Figure 2f shows a sharp gain decrease at 3.82 GHz and 8 GHz, which means good band-notch characteristic can be achieved. It can be seen from Figure 2f that the lowest value of gain is −8.06 dBi at 3.82 GHz, which means good out-of-band suppression can be achieved. The optimized geometrical parameters of the GAF antenna are shown as follows: W1 = 71 mm, W2 = 26.3 mm, W3 = 9 mm, W4 = 2 mm, L1 = 98.35 mm, L2 = 54 mm, L3 = 24.35 mm, L4 = 20 mm, L5 = 10.5 mm, L6 = 8.5 mm, L7 = 3.75 mm, D1 = 7 mm, R1 = 7.5 mm, Theta = 100°.

The distribution of antenna surface current has great influence on antenna radiation pattern. A new idea for antenna design can be provided by studying the distribution of surface current. To explore the mechanism of band-notch antenna, the surface current distributions of the GAF antenna at different frequencies are illustrated in Figure 2g–i. In Figure 2g, the SRR and OCHWR have ignorable impact on the resonance characteristic of GAF antenna at 2.45 GHz. On the other hand, as shown in Figure 2h, the SRR is equivalent to a resonator, which has a big resonance effect on the antenna at 3.8 GHz [30]. Most of the surface current energy is concentrated on the SRR and the antenna almost cannot radiate. In the same way, when the antenna operates at 8 GHz, most of the surface current energy are concentrated on the OCHWR. Thus band-notched characteristics can be achieved. The notch frequency can be adjusted flexibly by adjusting the size of notch unit.

### 3.2. Conformal Characteristics of GAF Antenna

**Figure 3:**
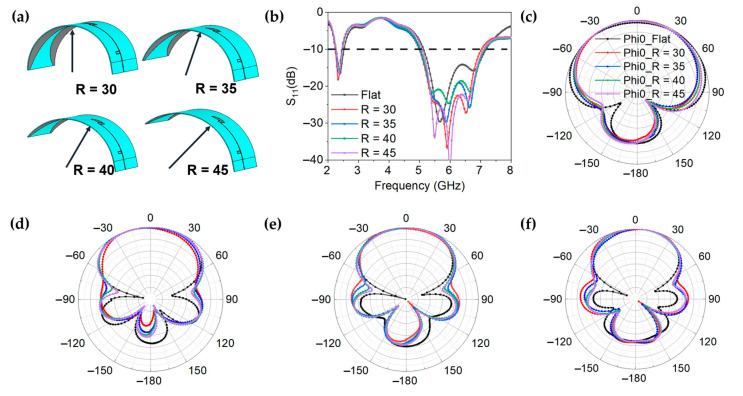
(**a**) Proposed GAF antenna under different bending angles. (**b**) Simulated reflection coefficients of the GAF antenna under different bending states. (**c**–**f**). Simulated radiation patterns of the GAF antenna under different bending radii at different frequencies of (**c**) 2.45 GHz. (**d**) 5.5 GHz. (**e**) 6.0 GHz. (**f**) 6.5 GHz.

For conformal antennas, it is important to keep the resonant characteristics stable in bending state. To explore the resonance characteristics of GAF dual-band antenna in the bending state, a conformal experiment is designed, as shown in Figure 3a. The bending radius of the antenna is changed to simulate different conformal conditions. The GAF dual-band antenna is bent with the bending radius R of 30 mm to 45 mm, and the corresponding bending angle is 180° to 120°.

Figure 3b shows the simulated results of resonance characteristics of GAF dual-band antenna under different bending states. Although the equivalent electric length is increased while the antenna is bent, and the resonant frequency of the antenna is reduced, the working bandwidth of GAF dual band antenna is almost unchanged, even at a bending angle of 180°, which indicates that a flexible GAF antenna can cover the vast majority of current conformal application scenarios. In addition, the simulated far-field radiation patterns under different bending angles are shown in Figure 3c–f. It can be clearly seen that the radiation consistency of the GAF dual-band antenna is good in a series bending state in the whole working frequency band. These results show that the resonant characteristics of the designed antenna can be kept stable under different application scenarios.

### 3.3. Fabrication and Measurement of GAF Dual-Band Antenna

**Figure 4:**
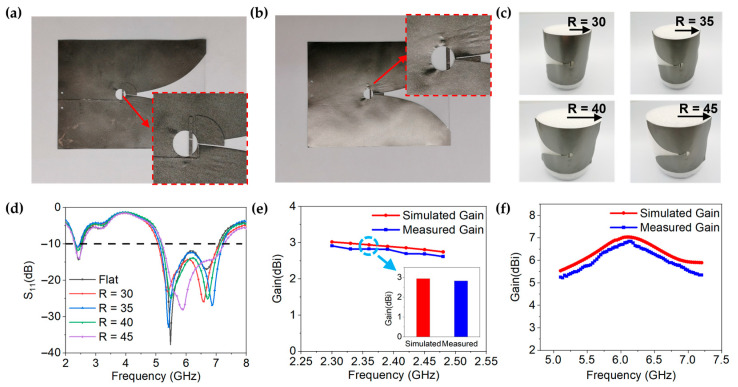
(**a**,**b**) Digital photos of the GAF dual-band antenna. (**a**) Top view. (**b**) Bottom view. (**c**) Digital photos of conformal antenna under different bending angles. (**d**) Measured reflection coefficients of the GAF antenna under different bending states. (**e**,**f**) Simulated and measured realized gain of the GAF antenna.

The GAF antenna is fabricated by a one-step laser-direct mold engraving method. The model of the laser engraving machine used is LPKF ProtoLaser S, and the resolution is 25 μm. The PDMS substrate is then machined by a mechanical cutter bar to obtain the desired size. Finally, the graphene conductive layer and the PDMS dielectric layer are assembled to obtain the GAF conformal antenna, which is shown in Figure 4a,b. The thickness of the antenna is only 0.548 mm, which means a low profile is achieved.

As shown in Figure 4c, the GAF antenna is conformed on foam cylinders with different radii of 30 mm–45 mm to explore the resonance characteristics in different conformal states. The relative dielectric constant of foam cylinders is 1.05, which has little effect on antenna radiation. The feeder port of antenna was connected to a SMA-KHD 23 connector, which was linked to a power network analyzer (PNA) through a coaxial line. The reflection coefficients of the antenna were measured by the PNA. The radiation patterns and gain of the antenna were derived from the antenna measurement system, which consisted of a PNA, a diamond engineering antenna measurement system (DAMS) platform controller, and a microwave anechoic chamber. The PNA was connected to a positioner platform and a standard reference antenna (REF antenna) of the microwave anechoic chamber to control signal reception. The DAMS platform controller was connected to the positioner platform of the microwave anechoic chamber. The radiation patterns were obtained by a rotating antenna under test (AUT) with increments of 3°. Figure 4d is the measured resonant frequency and return loss of the GAF dual-band antenna. In the bending radius of 30 to 45 mm (the corresponding bending angle of 180°–120°), the GAF dual-band antenna can cover 2.40–2.45 GHz and 5.15–7.1 GHz, although the resonant frequency is slightly shifted. Therefore, GAF antenna can work normally, even under bending angle of 180°.

**Figure 5:**
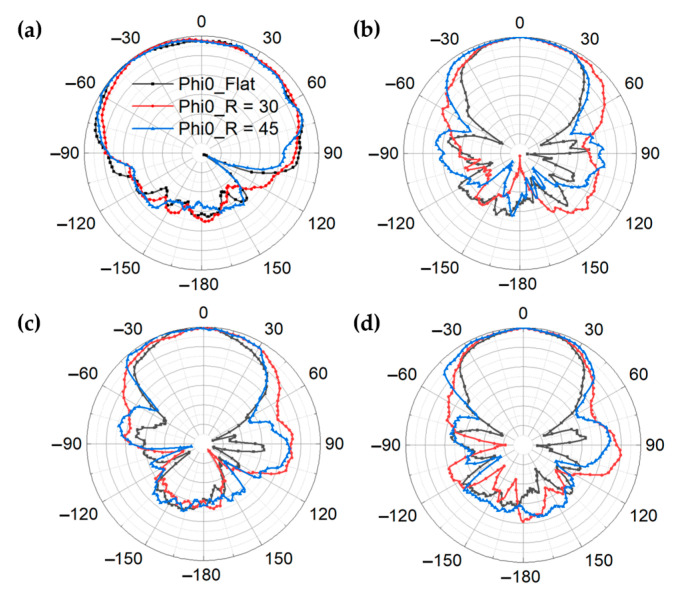
(**a**–**d**) Measured radiation patterns of the bent GAF antenna at different frequencies of (**a**) 2.45 GHz; (**b**) 5.5 GHz; (**c**) 6.0 GHz; (**d**) 6.5 GHz.

As demonstrated in Figure 4e, f, the simulated and measured realized gain of GAF antenna show good consistency. The measured realized gain ranges from 5.25–6.85 dBi within 5.1–7.2 GHz and 2.62–2.91 dBi within 2.3–2.5 GHz. Figure 5a–d show the measured radiation patterns of GAF dual-band antenna at 2.45 GHz, 5.5 GHz, 6.0 GHz and 6.5 GHz in different conformal states. Although the radiation patterns are slightly distorted after bending, the radiation consistency is still good in the whole frequency band.

**Figure 6:**
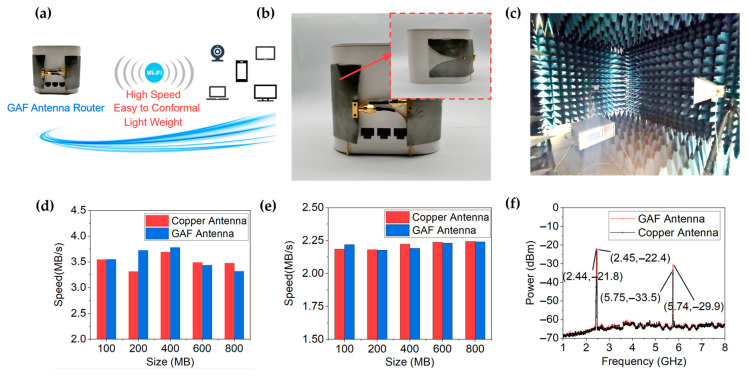
(**a**) Schematic diagram of application scenarios of GAF antenna router. (**b**) Digital photo of the fabricated GAF antenna conformed to a commercial router. (**c**) Photograph of the measurement environment. (**d**) Measured upload speeds of the routers with GAF antenna and copper antenna. (**e**) Measured download speeds of the routers with GAF antenna and copper antenna. (**f**) Measured spectrum power of the routers with GAF antenna and copper antenna.

As shown in Figure 6a, the proposed GAF antenna is used in the router, which has the advantages of high transmission rate, easy to conformal and light weight. Figure 6b depicts that the GAF antenna router is fabricated by replacing the original copper antenna of the router with the GAF antenna. By conforming the GAF antenna to the surface of the router, space utilization can be improved and miniaturization can be achieved. The transmission rates and spectral power of the GAF antenna and copper antenna were measured in an anechoic chamber as shown in Figure 6c. It can be seen from Figure 6d,e that the GAF antenna has a transmission rate comparable to that of a copper antenna. Figure 6f shows that the spectral power of the GAF antenna is greater than the original copper antenna in the router, which means a higher signal-to-noise ratio and a longer transmission range can be achieved.

## 4. Conclusions

In conclusion, a flexible GAF dual-band antenna with low profile and light weight is designed, fabricated and measured. The GAF antenna possesses the mechanical advantages of good flexibility and excellent flexural endurance. The designed GAF antenna has a low profile of 0.548 mm, which is very suitable for conformal applications. The measured peak realized gain of 6.85 dBi is obtained at 6.12 GHz. Furthermore, the measured results show that the GAF antenna has good matching performance and stable radiation patterns in the frequency bands of 2.4–2.45 GHz and 5.15–7.1 GHz, even under the bending angle of 180°. The fabricated GAF antenna router has the advantages of miniaturization, light weight and high transmission rate. Furthermore, the spectral power of the GAF antenna router is greater than the copper antenna router, indicating a higher signal-to-noise ratio and longer transmission range. All the results show that GAF dual-band antenna has great application potential in the next generation of conformal Wi-Fi antenna.

## Data Availability

Not applicable.
